# Novel approaches for Spatial and Molecular Surveillance of Porcine Reproductive and Respiratory Syndrome Virus (PRRSv) in the United States

**DOI:** 10.1038/s41598-017-04628-2

**Published:** 2017-06-28

**Authors:** Moh A. Alkhamis, Andreia G. Arruda, Robert B. Morrison, Andres M. Perez

**Affiliations:** 1 Department of Veterinary Population Medicine, College of Veterinary Medicine, University of Minnesota, St. Paul, USA; 2 Faculty of Public Heath, Health Sciences Center, Kuwait University, Kuwait City, Kuwait; 3 Department of Veterinary Preventive Medicine, College of Veterinary Medicine, The Ohio State University, Columbus, USA

## Abstract

The US swine industry has been impaired over the last 25 years by the far-reaching financial losses caused by the porcine reproductive and respiratory syndrome (PRRS). Here, we explored the relations between the spatial risk of PRRS outbreaks and its phylodynamic history in the U.S during 1998–2016 using ORF5 sequences collected from swine farms in the Midwest region. We used maximum entropy and Bayesian phylodynamic models to generate risk maps for PRRS outbreaks and reconstructed the evolutionary history of three selected phylogenetic clades (A, B and C). High-risk areas for PRRS were best-predicted by pig density and climate seasonality and included Minnesota, Iowa and South Dakota. Phylodynamic models demonstrated that the geographical spread of the three clades followed a heterogeneous spatial diffusion process. Furthermore, PRRS viruses were characterized by typical seasonality in their population size. However, endemic strains were characterized by a substantially slower population growth and evolutionary rates, as well as smaller spatial dispersal rates when compared to emerging strains. We demonstrated the prospects of combining inferences derived from two unique analytical methods to inform decisions related to risk-based interventions of an important pathogen affecting one of the largest food animal industries in the world.

## Introduction

The United States (U.S.) is the third largest pork-producer country and the second major exporter globally^1^. Porcine reproductive and respiratory syndrome (PRRS) first appeared in the southeastern region of the U.S. in 1989, causing reproductive failure in sows and respiratory disorders in pigs of all ages^2^. Subsequently, the disease spread throughout the North American swine population and became endemic. Although the disease was first discovered in the U.S., earliest serological evidence suggests that the virus first emerged in eastern Canada^3^. In 2011, the annual losses associated with PRRS virus (PRRSv) infections on the national economy of the U.S. reached up to USD 664 million^4^. Thus, arguably, the disease has become one of the most import swine diseases and has been considered a national threat to food security^5^. PRRSv is an enveloped single-stranded RNA virus and belongs to the *Arteriviridae* family^3^. The virus has seven structural and 14 non-structural proteins encoded by a genome that comprises nine open reading frames (ORF)^6^. Envelope surface glycoprotein (GP5) is encoded by gene segment ORF5, which is characterized by the highest genetic diversity compared to other ORF segments, and thus, became popular for most molecular epidemiology studies^7–9^.

PRRSV infections have been reported worldwide, except for Australia and Antarctica, and have been classified into two main groups; North American and European strains^10, 11^. The European strain is referred to as Type I genotype and has relatively low prevalence in the U.S. The North American strain is referred to as Type II genotype and is currently causing most PRRSv infections in the country^11^. PRRSv can be transmitted rapidly through direct and indirect contact^12^, which include between-farm transmission through animal movement (direct contact between infected and susceptible animals), fomites and airborne transmission^13, 14^. Endemic PRRSv strains are commonly characterized by annual seasonal increases in the number of observed outbreaks, with incidence of cases being low during spring and summer and high during fall and winter^15^. However, the emergence of new virulent viruses is also a common characteristic of PRRSv (referred to as emerging strains), with “spreader events” being recognized for different United States regions^16^; those emerging strains are of considerable concern to the swine industry as they cause severe economic losses. It is currently unknown if alternative transmission routes are equally important for emerging and endemic PRRSv strains.

Currently, PRRSv control and prevention activities are unregulated in the U.S., which, combined with the varied and rapid biological properties of this RNA virus and the limited effectiveness of available vaccines, jeopardize the effectiveness of those mitigating measures^17^. Hence, risk-based interventions against emerging strains are required to minimize their impact on the industry^5^. Current swine production systems are commonly not separated by well-defined geographical boundaries and are characterized by highly frequent, often long-distance movement of pigs and supplies. Indeed, geographical distribution and dynamics of PRRSv spread are influenced by system-level decisions and needs, as well as environmental risk factors including, mainly, pig density and climate^18–20^.

Species distribution models (SDM) are spatially explicit analytical methods that offer the possibility to build predictive risk maps using disease presence and environmental data^21^. SDM are capable of extracting associations between the presence of disease cases (or outbreaks) and environmental factors to characterize environmental requirements for pathogen occurrence, which subsequently provide predictions on suitable geographical locations for virus circulation and spread over non-sampled areas^21^. SDMs have recently become popular for risk mapping of both animal and human diseases^22–25^ at local, regional and global scales. Such methods have been proven useful for modeling spatial distribution of diseases over large geographical areas, and thus, can provide a supportive platform for targeted sampling schemes of PRRSv within and between swine production regions in the U.S.

Molecular characterization of PRRSv constitutes a substantial portion of the pathogen’s surveillance efforts in the U.S., where a large number of sequences became available due to the growing accessibility to affordable molecular tests^26^. However, the increase in the size of PRRSv’s genomic data led to new challenges regarding the interpretation of results, especially considering the rapid mutation and recombination events of the virus. One of those challenges relates to the need for differentiating endemic from emerging strains. Past PRRSv evolutionary epidemiology studies either focused on establishing associations between phylogenetics and outbreak characteristics in different geographical levels^7, 27–29^, or discriminating between endemic and emerging strains to infer about their spread and maintenance within affected swine populations^7, 10, 30–32^. Those studies relied on traditional phylogenetic methods to either genotype new viruses using the restriction fragment length polymorphism (RFLP) patterns, or assess correlations between the similarities of nucleotide sequences and spatio-temporal outbreak dynamics^33^. Such methods ignore important evolutionary parameters, uncertainties associated with phylogenetic relationships, and spatio-temporal factors that shape the evolutionary history of rapidly evolving pathogens like PRRSv^34, 35^. Thus, tools provided by the field of phylodynamics have become a necessity to effectively characterize the joint evolutionary and epidemiological patterns of rapidly evolving pathogens^36^. These methods heavily rely on Bayesian statistical frameworks, which can provide methods that are able to account for uncertainties in the evolutionary parameters of the phylogeny, and subsequently can provide estimates on population dynamics, divergence times, and history of geographic spread^37^.

In the past decade, a few studies attempted to reconstruct the evolutionary history of PRRSv using Bayesian phylodynamic models^14, 33, 38–40^. Such studies answered important a long-standing hypothesis on the evolutionary epidemiology of PRRSv, and subsequently encouraged the notion for such methods to be routinely applied in surveillance of field data with the ultimate goal of supporting risk-based interventions. However, following the continuous rapid growth in both size and complexity of PRRSv data, routine implementation of such analytical methods is quite challenging. To date, no analytical pipelines were formulated to rigorously distinguish between endemic and emerging PRRSv strains. Instead, the distinction between such strains is naively made, for example, through the incidence of outbreaks and traditional phylogenetic methods. The application of more robust analytical pipelines that specializes in characterizing spatiotemporal dynamics of PRRSv strains can be of great value for the swine industry by improving surveillance, and subsequently control and prevention measures.

In this study, we illustrate the prospect of combining inferences derived from modern spatial explicit and phylodynamic disciplines for routine surveillance of PRRSv, and, potentially, other food animal pathogens, with the goal of supporting risk-based interventions in near-real time. Specifically, the objectives of this study were to identify environmental requirements for the circulation and spread of endemic PRRSv strains, and to characterize the evolutionary features of endemic and emerging PRRSv strains in a complex geographical setting. Results here will ultimately help support prevention and control of the most devastating disease affecting one of the largest food animal industries worldwide.

## Results

**Figure 1:**
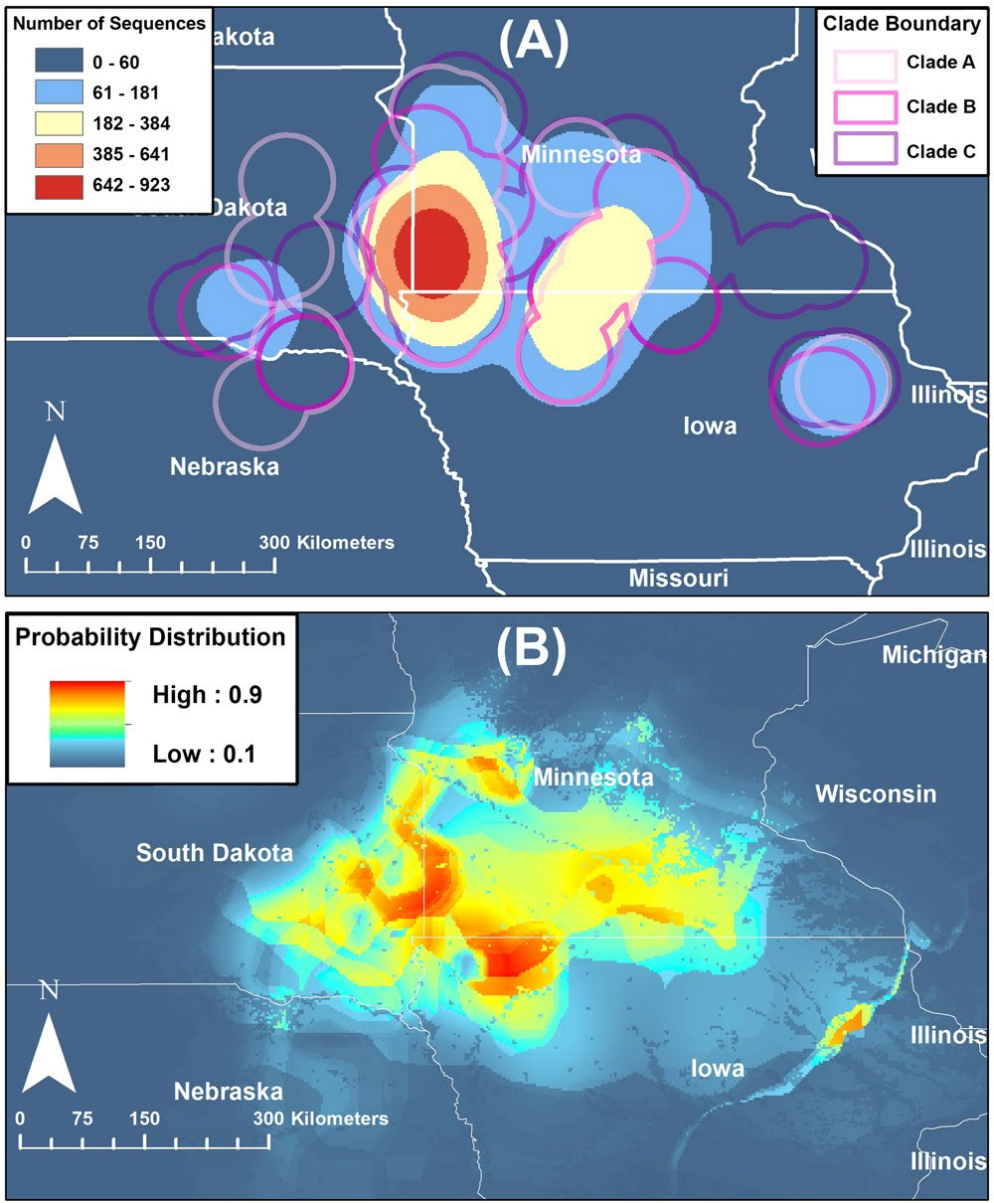
Geographical locations of the PRRSv sequences and predicted spatial probability of PRRSV outbreaks in swine farms located in the Midwest region of the U.S., collected from January 1998 to April 2016. (**A**) Smoothed kernel density function for the geographical locations of the farms where sequences were collected and geographical exntension of the selected clades. (**B**) Spatial probability distribution of PRRSV outbreaks predicted by the final presence-only maximum entropy ecological niche model. Figure’s maps were generated using ArcGIS version 10.4.

All PRRSv ORF5 sequences (n = 3,582) were field isolates obtained from different type of farms from one swine production system, including farrow to wean, farrow to feeder, and growing pig farms. The complete ORF5 nucleotide sequences were collected between 1^st^ January 1998 and 21^st^ April 2016 from PRRSv-infected swine farms located in the Midwest region of the U.S. Here, we defined the Midwest region to include Minnesota, Iowa, South Dakota and Nebraska. Sequencing was performed in Midwest-based veterinary diagnostic laboratories or private laboratories on a fee-for-service basis and according to the procedures in use at the time of virus detection. The information contained in the dataset obtained from the system included RFLP genotype classification, geographical location (latitude/longitude) of the farm from which the virus was isolated, and date of sequencing. The data were shared under a strict confidentiality agreement, in which identity of the production system and location of the participant farms cannot be revealed. Therefore, we generated and plotted a kernel density function of 5 km^2^ spatial resolution to represent the locations of infected farms (Fig. 1A) using ArcGIS version 10.4^41^. Sequence dataset used here is currently under preparation to be deposited in the Genbank and will be available soon to the readers with accessions xx-yy. Furthermore, full alignment of sequences has been provided on a FASTA file with the supplementary materials (File S1).

We used presence-only maximum entropy, an SDM technique (Maxent)^42^ to predict the geographical range of PRRSv high-risk areas in the Midwest region. Geographical locations of all sequences and a set of selected environmental predictors (Table S1) were used for the subsequent Maxent analyses. Furthermore, we used Bayesian Phylodynamic methods implemented in the BEAST^43^ software package to model the demographic and phylogeographic history of 3 selected viral clades, from the sequences described above. We decided to select three clades as cases studies, because analyzing 3,582 sequences at one run using complex and advanced phylodynamic methods is computationally demanding and time consuming, which was neither feasible for us nor the swine producers. The selected three clades were genetically distinct, in which two of them were dominated by strains known as endemic (based on the RFLP classification), whereas the third clade compromised emerging strains. Resulting risk maps and posterior parameters of the spatiotemporal diffusion history, from the Maxent and phylodynamic analyses, respectively, of the selected clades were combined visually to qualitatively infer some epidemiological insights about the characteristics of the selected PRRSv clades in the Midwest.

The set of selected environmental variables (Table S1) used in the Maxent analyses, included: (1) pig density; (2) climate; and (3) land cover. The density of pig farms in the region (referred to as pig density) was represented using a kernel density function with a spatial resolution of 5 km^2^ and was derived from locations of all pig farms across the US in 2012, retrieved from the United States Department of Agriculture Census of Agriculture website^44^. We obtained climate data (referred to as Bioclime) from the WorldClim in the form of 5 km^2^ resolution rasters^45^. We used 15 bioclimatic variables, out of the 19, and excluded the rest due to known spatial artifact in those four variables, as suggested elsewhere^46^. Finally, we retrieved MODIS-based global land cover climatology from the United States Geographical Survey (USGS) webpage, with a spatial resolution of 0.5 km^2^, to provide an estimate of the geographical distribution of 16 different land cover features in the US^47^. We processed the 17 environmental variables using the ‘Raster’ package^48^ implemented in R statistical software version 3.2.2^49^. All environmental layers were converted into a common projection, spatial resolution, and map extent, and each raster was cropped so that the geographical extent of the spatial analyses covered the Midwest swine production region. Raster data were aggregated and resampled to create a uniform grid size, which resulted in a scale of approximately 5 km^2^. Finally, we visually inspected collinearity between each pair of environmental predictors using scatter-plots.

### Species distribution modeling (SDM) of PRRSv spatial risk in the Midwest

Only four environmental predictors contributed to the geographical range of high-risk areas for PRRSv outbreaks in the Midwest, with AUC values greater than 0.7 (Table 1). Pig density was, by far, the most important environmental predictor, followed by temperature seasonality, precipitation seasonality, and land cover in the Midwest (Table 1). Areas with high pig densities, temperature changes around 11 to 13 °C, standard deviation and precipitation variability between 40% and 60% over the course of the year, and located nearby permanent wetlands were found mostly suitable for PRRSv outbreaks in the Midwest. Most PRRSv outbreaks (>25%) were observed in southwestern Minnesota and eastern South Dakota (Fig. 1A). However, the predicted spatial range of high-risk areas (Probability >0.8) in the Midwest included southern and western Minnesota, northwestern Iowa, and eastern and central of South Dakota as well (Fig. 1B).

**Table 1:** Estimates of relative contributions of environmental variables to the final Maxent model and their area under de curve (AUC) values.

Variable	% Contribution	Training Data AUC^a^	Test Data AUC ± SD^b^	cAUC^c^ ± SD
Pig Density	60.0	0.96	0.94 ± 0.08	0.75 ± 0.11
Temperature Seasonality	27.9			
Precipitation Seasonality	6.7			
Land Cover	5.4			

^a^Area under the curve. ^b^Standard deviation. ^c^Caliberated AUC.

### Demographic and Phylogeographic history of PRRSv in the Midwest

**Figure 2:**
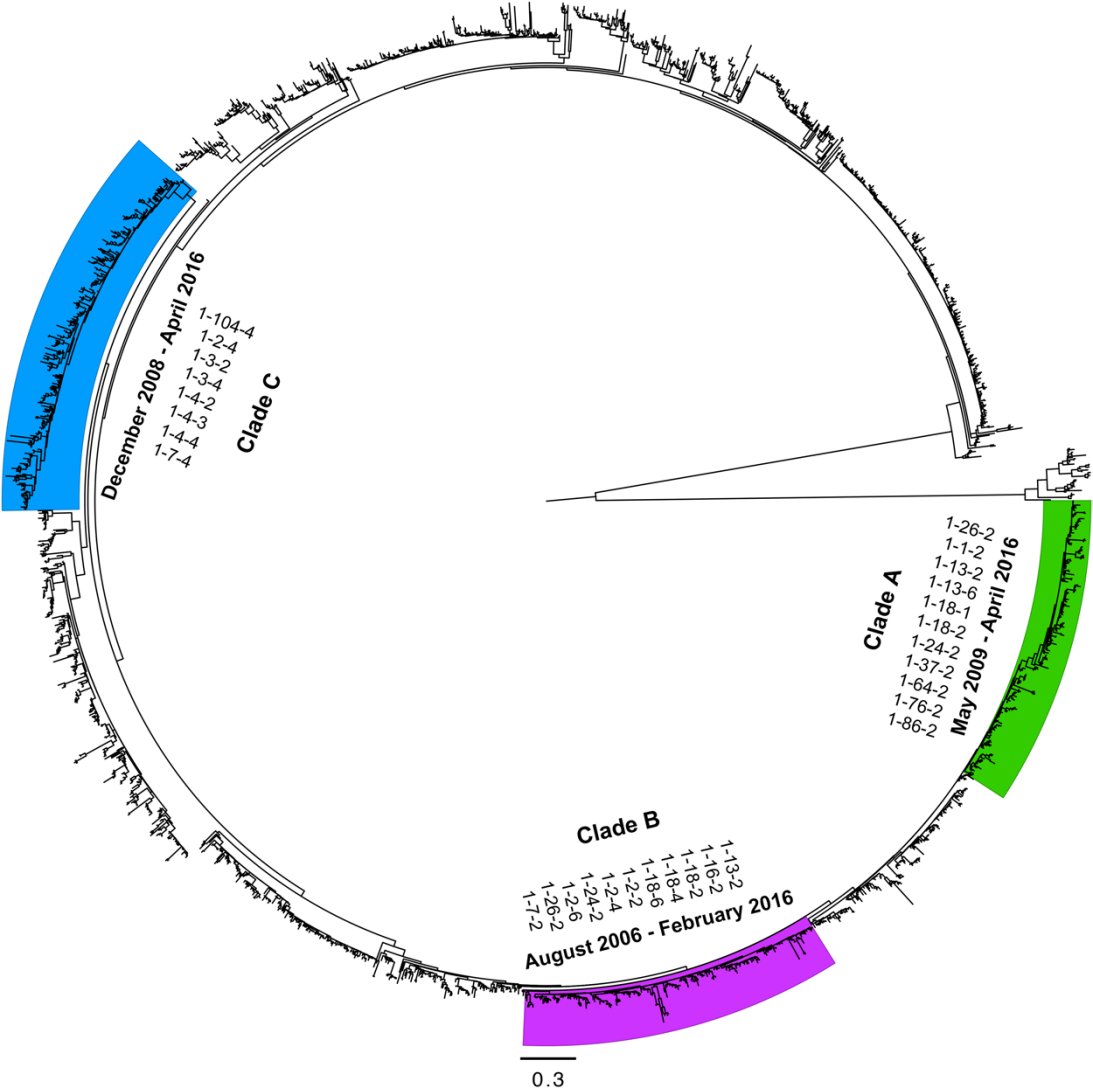
Maximum Likelihood (ML) phylogeny of PRRSV ORF-5 sequences collected between January 1998 and April 2016 in swine farms in the Midwest of the U.S. The ML tree was inferred from the GTR + Γ model of evolution. Support given at nodes based on through bootstrap search using 10 runs, with 100 ML replicates in each run, implemented in RAxML version 8. The scale bar indicates the number of substitutions per site. Selected clades are highlighted with green (Clade ‘A’), purple (Clade ‘B’), and Blue (‘F’), with their corresponding RFLP-types isolates listed and collection dates (Month-Year).

**Figure 3:**
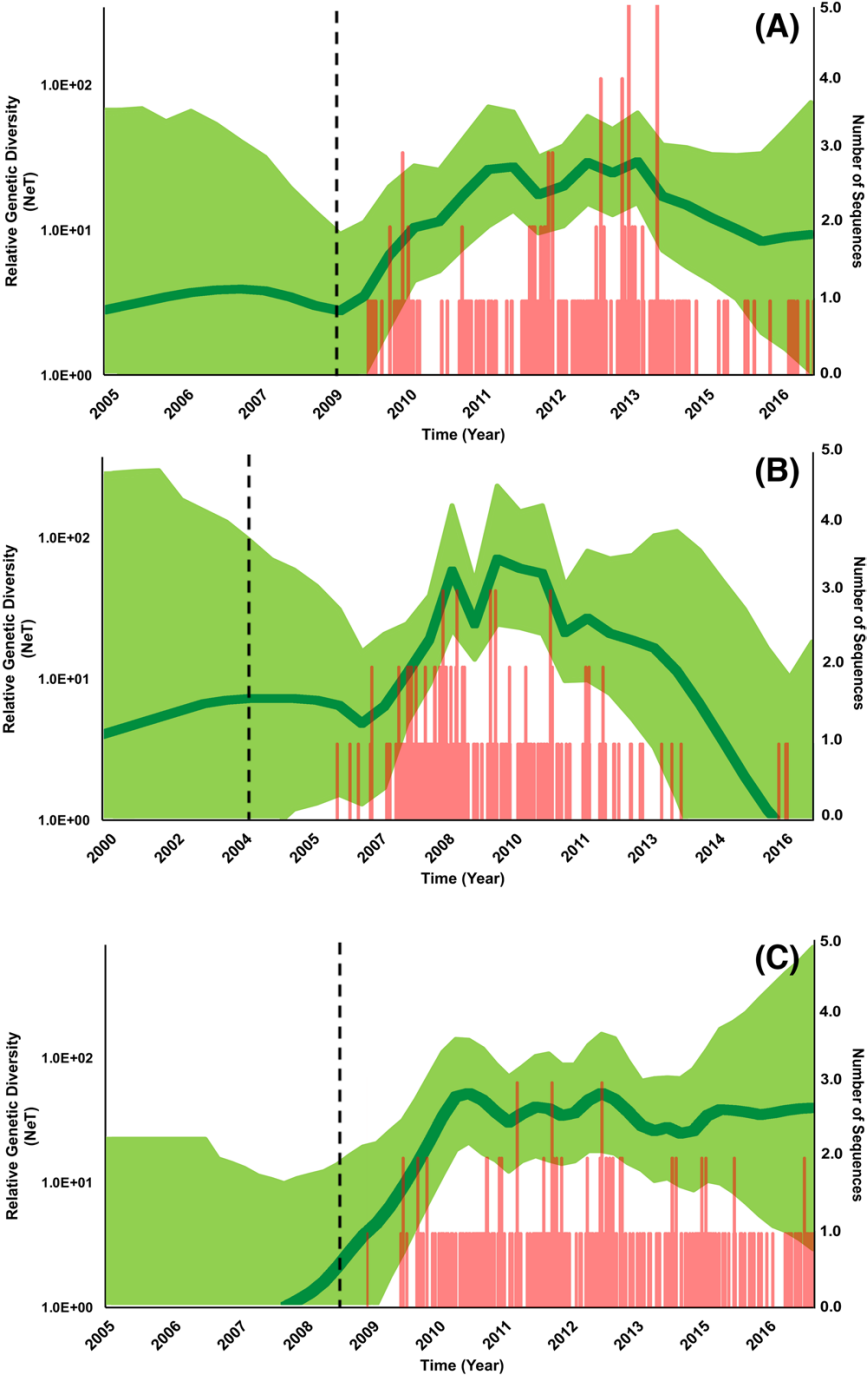
Temporal variation in the effective population size of PRRSv selected clades in the in the Midwest isolated between 2009 and 2016. This ‘Bayesian skygrid’ plot summarizes the inferred effective population size (*N*_*e*_ *T*)—which summarize genetic variation in terms of effective population size trajectories of the sampled ORF-5 gene sequences—through time in the Midwest. The mean estimate is indicated by the dark green line; the shaded light green regions correspond to the 95% HPD. Red bars represents the number of sequences collected over the period of the study and correspond to each selected clade. Vertical doted lines represent corresponds to the estimated time at which each clade transitioned from slow to fast population growth. Each figure (**A**–**C**) corresponds to Clades (**A**–**C**), respectively.

Results of the ORF5 Maximum likelihood (ML) tree yielded over 20 distinct PRRSV clades (Fig. 2). We selected three clades (A, B and C) for further analyses, ensuring that each selected clade’s spatial and temporal distribution represented the geographical extent and period of the study (Figs 1A and 2). Clades A and B ORF5 sequence data favored the expansion and the exponential coalescent tree models, respectively (BF > 20; Table S2). That finding suggests that Clade A viral population grew at an increasingly exponential rate, whereas Clade B population grew at a fixed exponential rate. However, while both clades favored different coalescent tree models, inferred posterior growth rate parameter was similar and equal to 0.23 year^−1^ (95% HPD:0.07, 0.38) and 0.21 year^−1^ (95% HPD:0.08, 0.36) for clades A and B, respectively. Clade C ORF5 sequence data favored the logistic coalescent tree model (BF > 34; Table S2), which assumes that population size of the virus grew at a decreasing rate over time (Table S2). However, Clade’s C inferred posterior growth rate parameter was substantially larger than that of Clades’ A and B and was equal to 2.63 year^−1^ (95% HPD:1.28, 4.20). Furthermore, estimates of the clades’ relative genetic diversity through time suggest that all the three clades exhibited typical annual seasonal increases in population size (Fig. 3).

**Figure 4:**
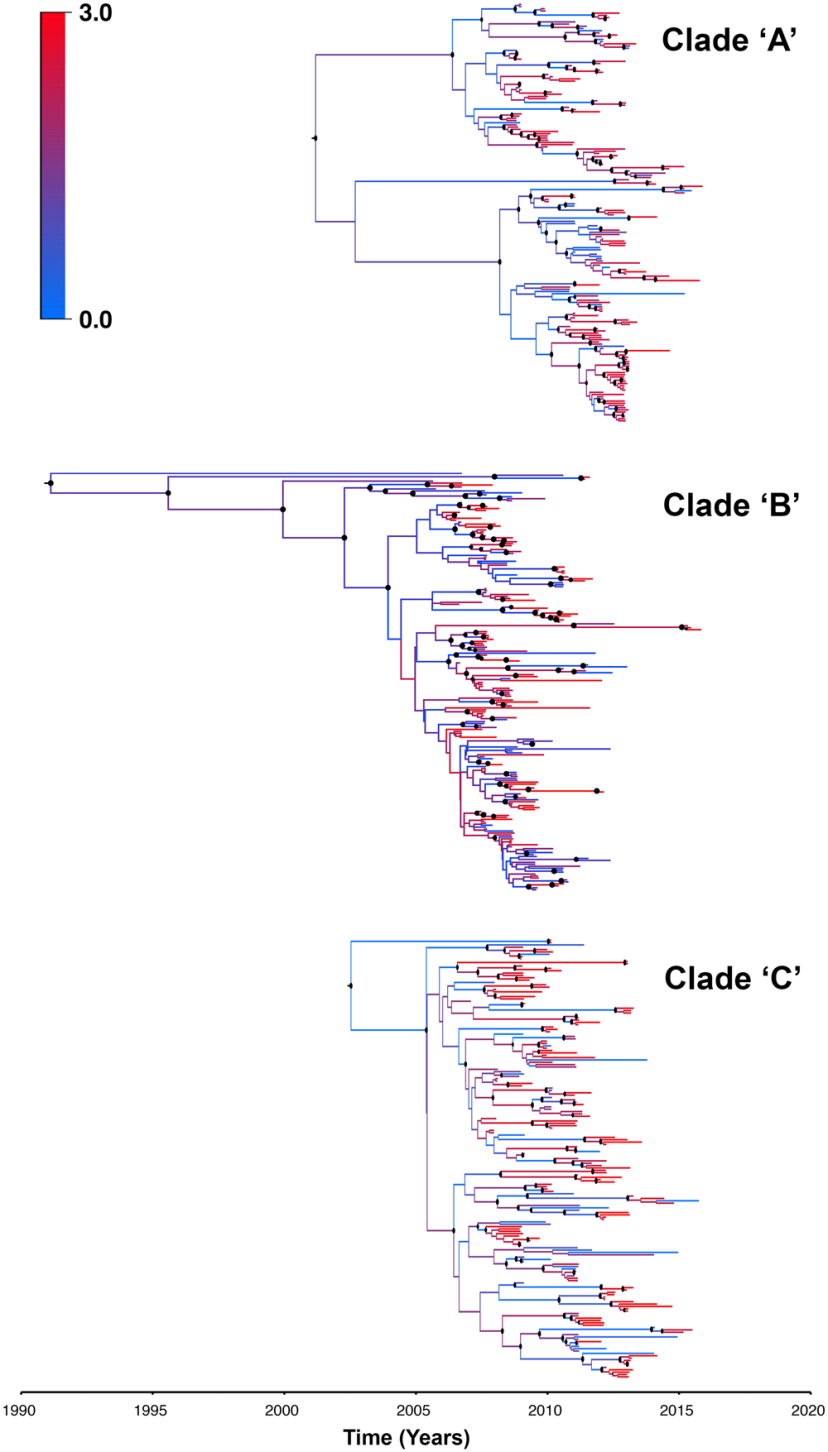
Maximum clade credibility (MCC) phylogeny of ORF-5 gene of PRRS clades ‘A’, ‘B’ and ‘C’ viruses in the Midwest estimated under the best-fitting spatial diffusion model (Table 2). The color of the branches represents the among-branch spatial diffusion rate variation and corresponds to the color gradient legend on the middle right. Well-supported posterior probabilities (P > 0.85) of branching events are indicated by black circles at the nodes.

Continuous diffusion phylogeographic models indicated that geographical spread of PRRSv outbreaks for the three clades followed a heterogeneous, rather than homogenous, spatial diffusion process (Table 2). Both A and B clades favored the gamma RRW model with initial dispersal rates of 16.3 and 13.1 km/year, respectively (Table 2). However, clade C favored the Cauchy RRW model with a dispersal rate that was substantially higher and reached up to 73.1 km/year (Table 2). Mean evolutionary rates of clades A and B were closely similar (0.0074 and 0.0089 substitution/site/year, respectively) and substantially smaller than clade C (0.0114; Table 2). However, viruses from clade C have evolved more recently than those from clades A and B (Table 2; Fig. 4). Tree topologies inferred by the ML method (Figure S1) for the three clades were substantially different from the topologies inferred by the Bayesian diffusion phylogeographic models (Fig. 4). For all the three clades, highest among-branch spatial diffusion rate variation was inferred at most of the tips of the MCC trees, which is a typical reflection of the heterogeneous diffusion process (Fig. 4).

**Table 2:** Posterior estimates of spatio-temporal evolutionary parameters and comparisons under different spatial diffusion models.

	Spatial diffusion model			
	Homogeneous diffusion	Heterogeneous diffusion		
		Cauchy	Gamma	LogNormal
Clade ‘A’				
ML by SS^a^	−5932.1	−5368.2	**−5229**.**5**	−5363.1
ML by PS^b^	−5929.5	−5362.9	**−5225**.**1**	−5363.8
In BF^c^	1408.8	278.2	**Best-fitting model**	277.4
Mean evolutionary rate	0.0072 (0.0057, 0.0087)	0.0084 (0.0068, 0.0101)	**0**.**0089** (**0**.**0072**, **0**.**0107**)	0.0082 (0.0066, 0.0098)
Date of outbreak origin	1999.7 (1996.8, 2002.7)	2001.7 (1998.8, 2004.1)	**2002**.**0** (**1999**.**4**, **2004**.**4**)	2001.4 (1998.7, 2004.1)
Dispersal rate (km/year)	23.8 (19.4, 28.4)	16.4 (13.8, 19.1)	**16**.**3** (**14**.**2**, **19**.**4**)	15.9 (13.6, 18.3)
Clade ‘B’				
ML by SS^a^	−6581.5	−6156.85	**−5991**.**9**	−6253.75
ML by PS^b^	−6576.8	−6151.25	**−5984**.**3**	−6250.95
In BF^c^	1185	329.8	**Best-fitting model**	533.3
Mean evolutionary rate	0.0066 (0.0055, 0.0080)	0.0073 (0.0061, 0.0086)	**0**.**0074** (**0**.**0061**, **0**.**0087**)	0.0073 (0.0061, 0.0087)
Date of epidemic origin	1989.1 (1984.0, 1993.8)	1990.4 (1985.4, 1995.2)	**1990**.**4** (**1985**.**4**, **1995**.**4**)	1991.0 (1986.1, 1995.4)
Dispersal rate (km/year)	14.6 (12.5, 17.1)	12.9 (11.2, 14.8)	**13**.**1** (**11**.**3**, **14**.**2**)	13.3 (11.4, 15.2)
Clade ‘C’				
ML by SS^a^	−6182.0	**−5097**.**9**	−5171.5	−5436.0
ML by PS^b^	−6185.2	**−5092**.**9**	−5169.4	−5406.5
In BF^c^	2184.6	**Best-fitting model**	153	627.2
Mean evolutionary rate	0.01 (0.0082, 0.012)	**0**.**0114** (**0**.**0094**, **0**.**0135**)	0.0117 (0.0098, 0.0139)	0.0116 (0.0096, 0.0135)
Date of epidemic origin	2005.5 (2004.5, 2006.4)	**2003**.**0** (**2000**.**7**, **2005**.**0**)	2006.4 (2005.6, 2007)	2006.2 (2005.4, 2007.0)
Dispersal rate (km/year)	39.3 (33.3, 45.2)	**73**.**1** (**59**.**2**, **87**.**4**)	61.4 (42.4, 85.5)	24.6 (21.7, 27.6)

^a^Marginal likelihood estimated by stepping-stone. ^b^Marginal likelihood estimated by path-sampling. ^c^Bayes factors.

**Figure 5:**
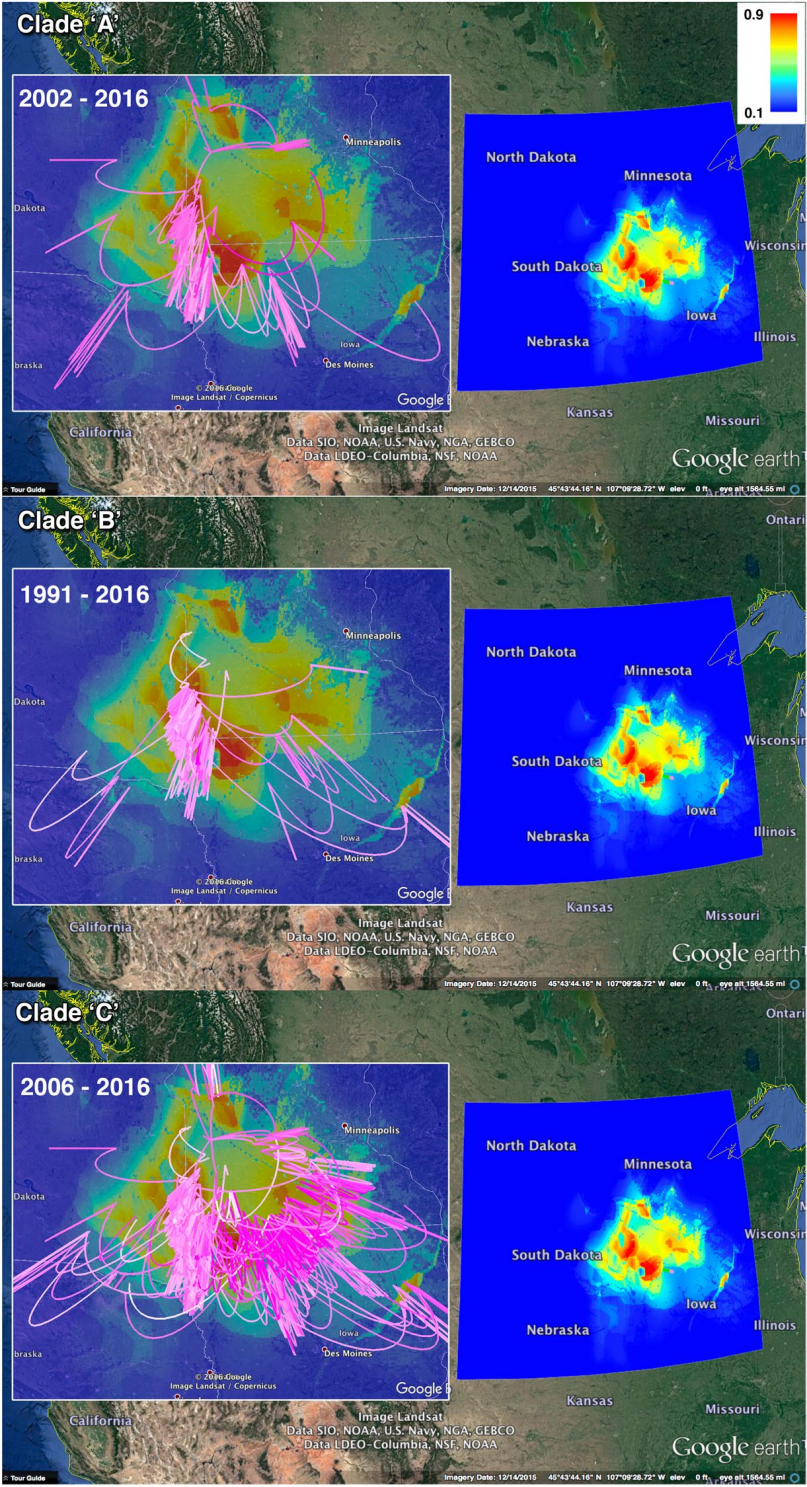
Clades’ ‘A’, ‘B’ and ‘C’ MCC tree spread and ecology in the Midwest. The Google Earth Pro snapshots of Clades MCC trees are superimposed on the predicted spatial risk of PRRSV outbreaks by the final Maxent model. The gradient color legend on the upper right represents the scale of the spatial probability distribution. The lines represent the continuous diffusion phylogeographic history of clade viruses, and their gradient colors represent their age (light pink = older, dark pink = younger). The figure is composed of satellite images captured as snapshots from Google Earth Pro (https://www.google.com/earth/). Figure’s associated three-dimensional movies corresponding to each clade are provided in the supplementary materials (File S2–4).

Finally, the inferred geographical origins of the posterior phylogeny and dispersal routes with a high rate of spatial diffusion for clades A and B were mostly encompassed by the identified high-risk areas predicted by the final Maxent model (Fig. 5). Indeed, most of the MCC tree branches of clades A and B had accumulated at the predicted high-risk areas (Probability <0.8), with narrow dispersal patterns (Fig. 5). However, even though clade C’s point of geographical origin was encompassed by the predicted high-risk areas in the borders between Minnesota and Iowa (Fig. 5), the MCC branches exhibited a substantially wider dispersal pattern compared to clades A and B, and did not accumulate in the predicted high-risk areas (Fig. 5). Instead, the MCC branches of clade C predominately spread throughout the study region as demonstrated in Fig. 5.

## Discussion

This study represents the first attempt to explore the potential of combining novel analytical methods including maximum entropy and Bayesian phylodynamic modeling to provide insights into the spatial and evolutionary epidemiology of viral diseases, using PRRSv in the U.S. a working example. We estimated, in quantitative and qualitative terms, the evolutionary history and extent of the association between environmental and demographic factors and geographical distribution of the PRRSv in endemic settings. We demonstrated that pig density and climate seasonality were not only important factors for maintaining endemic PRRSv strains, but also, they likely shaped genetic diversity over time as well as spatiotemporal diffusion patterns. Simultaneously, we revealed the wide and rapid spatiotemporal diffusion characteristics in the evolution of emerging strains. We demonstrated how the combined use of the analytical tools here was not only able to identify the geographical risk of PRRSv outbreaks but also were able to distinguish between endemic and emerging strains.

Although the highest incidence of PRRSv outbreaks (>%25) was observed in southwestern Minnesota and eastern South Dakota between 1998 and 2016 (Fig. 1A), our final Maxent model identified that the geographical risk of PRRSV outbreaks also included northwestern Iowa (Fig. 1B). The AUC values of the final Maxent model suggested that the selected environmental variables were adequate predictors for PRRSv outbreaks between 1998 and 2016 (Table 1). Pig density accounted for most of the background spatial risk in the Midwest (Table 1), as suggested elsewhere^5, 18, 19^. However, climate seasonality and land cover features had a relatively large role (approximately 40% relative contribution; Table 1) in predicting the risk of PRRSv outbreaks. Indeed, the combination of rainy and windy seasons, the large temperature variation over the course of the year, and proximity to permanent wetland, a dominant demographic characteristic of the Midwest, led to seasonal increases in the number of outbreaks, and contributed in maintaining the endemic state of PRRSv^50^. Despite these findings; it is important to point out that farms in the Midwest are mainly closed facilities where pigs are contained in a closed environment to minimize the impact of climate. Thus, the effect of climate and land cover may be due, at least in part, to climate- related anthropogenic activities, such as management practices, pig or semen movements and nearby farming activities, which occurs on seasonal patterns, as suggested elsewhere^5, 19^.

Results of the ORF5 gene BSg reconstruction revealed similar seasonal increases in the relative population size of the three PRRSv clades, which agrees with results from the final Maxent model described above and highlights the role of climate seasonality and climate-anthropogenic related activities in shaping the evolution of endemic viral strains. This finding, that area spread of PRRSv in high pig-dense regions (such as the Midwest) during certain environmental conditions (e.g. seasonal winds), has been previously suggested^38^. However, results from the spatial diffusion models (Table 2) for clades A and B showed that the transmission of the virus was heterogeneous rather than homogenous, suggesting that long-distance pig movements, climate- related anthropogenic activities, or other unexplored indirect networks (e.g. service providers) might play a larger role compared to aerosol transmission *per se* in dispersal of the virus across the Midwest^51, 52^. Our results are in agreement with past studies, which suggested that the shape of pig transportation networks within and between production regions have a significant role in shaping the phylogeny of the virus and mirrors the spatial diffusion of endemic strains^14, 33, 53^.

However, results from other phylodynamic analyses revealed distinct evolutionary characteristics of PRRSv isolated in the Midwest and could distinguish between endemic and emerging viral strains. For example, while all selected clades showed typical seasonality in the population size overtime, viruses of clades A and B favoured the expansion model (which assumes that population growth increases over time^54^), and exponential model (which assumes that population growth is fixed overtime^54^) (Table S2), viruses of clade C significantly favoured the logistic model (Table S2), which assumes that the population growth of the virus decreases over time^54^. In addition, the inferred growth rate for clades A and B was similar, but was 90% slower than that of Clade C. This result suggests that clade C was an emerging strain, which rapidly spread across the Midwest and then reached a certain equilibrium or died out. Indeed, the distinct fast growth rate (Fig. 3C) of emerging strains, such as those observed in clade C, accelerates their peak genetic diversity, which subsequently lead to population decline. Ecologically speaking, such observation may suggest that environmental conditions in the Midwest may accelerate the spread of emerging strains, ultimately leading to virus population decline due to the rapid decrease in the size of the susceptible population. Furthermore, clade C contained viruses that belonged to RFLP type 1-7-4 and some of its close relatives, which has been reported previously^33^ as an emerging virulent strain which caused severe outbreaks between 2014 and 2015 across the U.S. Moreover, clade C demonstrated a substantially larger mean evolutionary and dispersal rates than clades A and B (Table 2), suggesting a distinct evolutionary behaviour of an emerging PRRSv strain. Finally, Fig. 5 demonstrates the rapid and widespread of the MCC tree branches of clade C all other the Midwestern swine production system, and did not accumulate in the highest-risk areas predicted by the Maxent model, like clades A and B. This result suggests that clade C evolution and spread is not maintained by the environmental characteristics of the Midwest region.

Both clades A and B had similar inferred posterior dispersal rates (16.3 and 13.1 km/year, respectively; Table 2), suggesting that both clades share similar spatiotemporal evolutionary patterns. Additionally, our inferred MCC trees for both clades demonstrated initial low among-branch spatial diffusion rate variation at the early stages of the epidemic, followed by a substantial increase in the rate variation at the branches’ tips (Fig. 4). This observation is also supported by Fig. 5, where dispersal of the MCC trees’ branches was substantially more intense in the later years than earlier years, suggesting that the dispersal rate of endemic PRRSV strains might continuously increase onward, in parallel to the expanding pig population densities in the Midwest region over time. Figure 5 also demonstrates how most of the MCC trees’ branches are concentrated in, and encompassed by the highest risk areas (probability >0.7) predicted by the Maxent model, suggesting that those geographical areas provide sufficient environmental requirements for the circulation and maintenance of PRRSv endemic strains.

Similar to clades A and B, results from the spatial diffusion model suggest that clade C followed a heterogeneous spatial diffusion process (Table 2), indicating that most PRRSvs, circulating or emerging in the Midwest region, are most likely transmitted or maintained by geographically heterogenous activities. This conclusion is important, because airborne spread of this particular clade would have likely resulted on homogeneous dispersal. In contrast, the heterogeneous dispersal found here suggests that the clade spread by mechanisms other than airborne spread only. Furthermore, our inferred MCC trees for clade C demonstrated, similarly to the other clades assessed here, an initial low among-branch spatial diffusion rate variation, followed by a substantial increase in the rate variation at the branches’ tips at the later stages of the virus dispersal (Fig. 4). However, both mean evolutionary and dispersal rates were substantially larger compared to those inferred for clades A and B (Table 2). Such behaviour is consistent with the emerging nature of clade C, because herds with a population that is naïve against emerging strains would accelerate virus mutation within the farm and, the virus geographical dispersal rate due to a subsequent increase in the between-farm transmission rates, associated with animal transportation activities.

This study represents the first attempt of using continuous spatial diffusion models for inferring posterior phylogeographic dispersal rates for PRRSv endemic strains (Table 2, Fig. 4). Inferring phylogeographic dispersal rates can be a useful measure for modeling the unobserved patterns of virus evolution in space and time^44^, and hence, shed deeper insights onto the degree of severity of virus transmission and spread within a swine production system over time. We expect that the analytical framework presented here will set the scene for improved surveillance aimed for early detection of new virulent emerging PRRSv strains, and subsequently guidance for risk-based intervention strategies. Our analytical approach can be used in the future for viruses that may emerge and re-emerge within the swine and other food animal industries, such as circovirus^55^.

Although Bayesian phylodynamic methods became well established for rapidly evolving RNA viruses like PRRS^56, 57^, past PRRSv studies continued using traditional phylogenetic methods (e.g. ML trees) to characterize new emerging strains, without accounting for their related evolutionary parameters, spatial or temporal information^58–62^. Furthermore, they continued to use the RFLP patterns to genotype the newly detected strains and made unrealistic and unsupported conclusions about their origins, transmission and evolution. A comparison between the MCC trees of the three selected clades (Fig. 4) and the ML tree (Figure S1), shows substantial discrepancies between their topologies estimated by the Bayesian phylodynamic and ML methods, respectively. Furthermore, the MCC tree (Figure S2) demonstrates how the past RFLP pattern classification method based on the ML tree for PRRSvs was redundant and doesn’t correctly place newly detected viral strains in their truly related viral clade or cluster. In contrast, by accounting for important evolutionary and epidemiological information in our Bayesian phylodynamic models, we were able to estimate and quantify many inferences (Table 2) that distinguish between endemic and emerging strains and, therefore, we made realistic and data-supported conclusions about their evolutionary, spatial and temporal characters. Our analytical approach offers a realistic and robust framework for spatial inference from genetic data, when information about the specific geographical locations of the outbreaks (latitude/longitude) is available. Past studies^14, 33^ attempted to infer the phylogeographic history of the virus using discrete traits (i.e. countries or regions, instead of actual geographical locations), which doesn’t provide a realistic representation of the spatial diffusion process of the virus within a geographical region. Indeed, here we could not only provide a realistic representation of the spatiotemporal diffusion process of the virus within the Midwest, but also we were able to quantify the spatial dispersal rate of this diffusion process and distinguish between the phylogeographic evolutionary characteristics of endemic and emerging strains. Furthermore, we could, indirectly, establish relationships between the geographical suitability for endemic strains (which has been predicted spatial risk by Maxent model) and their evolutionary history. To our knowledge there is no valid quantitative method that can statistically link result outputs from both analytical methods. That said, we must acknowledge that the direct inclusion of the Maxent environmental predictors into the phylodynamic analysis might improve our posterior inferences about the evolutionary dynamics of the virus in the Midwest. Lemey *et* *al*., (2014) developed an extension for his novel Bayesian discrete phylogeographic inference^37^, that can include and exclude environmental variables into the phylodynamic model^56^, but the method is currently not optimized for continuous space phylogeography, as is the case of the study here. Optimizing the GLM method for continuous space phylogeography would be of great value for future studies as well as for molecular surveillance, as it yields more realistic inferences about the spatial and evolutionary epidemiology of rapidly evolving pathogens like PRRSv. Therefore, our study represents a motivation to pursue future studies related to establishing a full quantitative pipeline for combining the inferences derived from both analytical methods.

Another limitation of the present study was attributed to the lack of information related to spatiotemporal patterns of pig movements. Such information would substantially improve the inferences of the methods presented here. With the continuous advancements in the phylodynamic methods, described above, the inclusion of pig movement into future phylogeographic models of PRRSv will soon be possible as it would, indeed, yield a more realistic inference about the spatiotemporal evolutionary patterns of PRRSv endemic and emerging strains. Finally, data represented only a sample from the region, and data from other farms and systems may have helped to improve our predictions; noteworthy, however, the quantity of data available to us is unprecedented.

In conclusion, PRRS viruses are characterized by typical seasonality in their population size. However, endemic strains are characterized by a substantially slower population growth and evolutionary rates, as well as smaller spatial dispersal rates when compared to emerging strains. Thus, study here demonstrates an analytical framework for inferring the evolutionary history of strains that coincidently circulate in a region. The framework provided valuable inferences with application to disease control, including, for example, predictions on the likelihood that specific clades or strains will continue to spread, or not, and the most likely mechanism of spread, as suggested by the homogeneity of the dispersal pattern. While we were unable to provide a direct quantitative link between the analytical methods used in this study, the results presented here will help to inform decisions on disease surveillance and control for, arguably, one of the most important non-regulated diseases affecting one of the largest food animal industries in the world.

## Materials and Methods

### Species distribution modeling

The Maxent method is implemented as a function in the ‘Dismo’ package in R^63^. This method has recently become popular for predicting spatial distribution of infectious diseases of both public health and veterinary significance and has been described extensively elsewhere^64, 65^. We used the default logistic model, convergence threshold, regularization, and number of iterations. We used a Jackknife test to calculate the contribution of each environmental variable to the final Maxent model, and evaluated the performance of the candidate Maxent models by partitioning the data into training and testing sets and using the threshold independent method (TIm)^66^. We set the TIm k-fold partitioning scheme to create 5 partitions and randomly sample each partition with replacement, where our candidate Maxent model was tested five times (k = 5) against 10,000 randomly generated background points (pseudo-absences). Subsequently, we calculated the area under the curve (AUC) through a receiver operator characteristic (ROC) plot of the sensitivity vs. 1 - specificity over the whole range of threshold values between 0 and 1. We used training AUC for model building, whereas the testing sets to evaluate model accuracy using the average value of the AUC calculated for each partitioned set. AUC values >0.75 for both training and testing data were considered reliably discriminating models and indicated that the selected environmental layers were adequate predictors^67, 68^. Finally, because the geographic extent of the study area was fairly large, we used a calibrated AUC (cAUC) final Maxent model to evaluate the presence of the spatial sorting bias (SSB) as suggested elsewhere^69^. If the cAUC value was close to 1, then we concluded the absence of SSB (i.e. locations within the Midwest region with the highest number of observed outbreaks have a small impact on the resulted Maxent model), whereas if the value was close to zero, we concluded the opposite. We then plotted the spatial probability distribution of PRRSV outbreaks predicted by the final Maxent model (Fig. 1B) using ArcGIS version 10.4^41^.

### Preliminary phylogenetic analysis

We converted the collection date for each sequence into fractional years (decimal days) to estimate divergence times. Then, we aligned the ORF5 sequences using MUSCLE^70^ and confirmed the reading frame by examining the amino-acid translation using AliView (Lorenz *et al*., 2011). Finally, we found no homologous recombinant sequences using the Recombination Detection Program^71^. Next, we used PartitionFinder^72^ to define the partitioning scheme of our alignment corresponding to the codon positions of the ORF5 protein-coding gene and we selected the most realistic partition schemes for the set of substitution models implemented in BEAST 1.8.4^43^ for the subsequent analyses based on the value of Bayesian Information Criterion (BIC). Then, we estimated the maximum-likelihood (ML) of the phylogeny for all ORF5 sequences, using RAxML^73^, and examined the tree topology under the GTR + Γ substitution model, with 100 non-parametric bootstrap replicate searches. Finally, we selected three well-supported phylogenetic clades, referred to as clades A, B and C, and ensured that each selected clade’s spatial and temporal distribution well represented the geographical extent and period of the study (Fig. 2). Clade A compromised 191 non-identical sequences collected between May 2009 and April 2016, clade B compromised 214 non-identical sequences collected between August 2006 and February 2016, and clade C compromised 187 non-identical sequences, collected between December 2008 and April 2016. Again, we performed RAxML analyses on each selected clade to compare the topology of the ML tree to the corresponding estimated topology of the posterior phylogeny in the subsequent Bayesian analyses (Figure S1).

### Model selection and estimation of virus evolutionary demographics

We inferred posterior phylogenetic relationships and demographic for each of the three PRRSv clades from the alignment of the sampled ORF5 sequences within a Bayesian statistical framework using relaxed-clock models implemented in BEAST 1.8.4^43^. We used a 3-codon position mixed GTR + Γ substitution model to realistically represent the sequence data for all the three clades (as indicated by the value of the BIC using ParitionFinder). Next, we evaluated five tree prior models for each of the three clades under a separate analytical setting, including (1) the constant population size (CP)^74^; (2) the exponential growth (EG)^45^; (3) the expansion growth (EGx)^54^; (4) the logistic growth (LG)^54^ parametric coalescent models; and (5) the nonparametric, piecewise-constant Bayesian Skygrid (BSg) coalescent model, which employ a Gassian Markov Random Field (GMRF) prior to smoothing the trajactories of the past population dynamics (BSg-GMRF)^75^. Furthermore, for each tree prior model, we evaluated two branch-rate models using: (1) the uncorrelated lognormal (UCLN)^76^ branch-rate prior model, and; (2) the uncorrelated exponential (UCED)^76^ branch-rate prior model. We estimated parameters of the branch-rate prior distributions using the continuous-time Markov chain (CTMC)-rate reference 77 as a hyperprior for the mean of the lognormal and exponential distributions, and an exponential hyperprior for the standard deviation of the lognormal distribution. Isolation dates of the sequences were used to calibrate tree-height (divergence times) estimates. We estimated the marginal likelihood of each candidate relaxed-clock models (ten candidate models for each of the three clades) using path-sampling (PS)^77, 78^ and stepping-stone sampling (SS)^79–81^ estimators. We estimated the posterior evolutionary parameters, phylogeny and population demographics under each candidate relaxed model using two replicate MCMC simulations for 200 million cycles and sampled every 20,000^th^ state, to assess the stability of the marginal-likelihood estimates. We used Tracer v. 1.6^82^ to assess the reliability of the MCMC simulations by examining the convergence of each MCMC simulation to the stationary distribution and calculating effective sample size (ESS)^83^ for every parameter. We assessed mixing of each chain over the stationary distribution by monitoring the acceptance rates for all parameters. We then evaluated the fit of each candidate relaxed-clock models based on the resulting marginal-likelihood estimates using the Bayes factor (BF) comparison approach (Table S2). Finally, we used the best-fitting branch-rate prior (UCED *v*.*s*. UCLN) with the BSg-GMRF coalescent model (Table S2) to generate a BSg plot of the effective population size trajectories between 1998 and 2016 for each PRRSv clade.

### Relaxed random-walk phylogeographic analyses

We extended the above BEAST analyses to infer the spatio-temporal evolutionary history of each clade using the continuous phylogeographic diffusion models, suggested by Lemey *et* *al*.^84^. This approach models movement of the virus in two dimensions as a scaled-mixture generalization of a Brownian motion process^83, 84^. Briefly, the method implements a diffusion rate variation by rescaling the diffusion process along each branch of the inferred posterior phylogeny^83, 84^. This procedure is achieved through scalers drawn from a specific distribution, which generates a scaled mixture of a wide range of relaxed-random walks. We used the priors of the best-fitting demographic models (coalescent tree prior/branch rate combinations), from the above analyses (Table S2), and extended the analyses using additional, independent runs to assess for the best continuous trait model that accommodate among-branch diffusion rate variation for each selected viral clade. This procedure included additional simulations (in duplicates to assess the stability of the resulting marginal-likelihoods) to evaluate the four continuous trait models implemented in BEAST, which included the simple naïve homologous Brownian model, and three heterogeneous relaxed random walks (RRW) models (Cauchy, gamma and lognormal probability distribution). We ran the subsequent MCMC simulations for 500 million states and sampled every 50,000 states. We used the resulting marginal-likelihoods, estimated by the PS and SS methods, to selected the best-fitting continuous trait model for each viral clade by performing additional BF comparisons.

We used TreeAnnotator to summarize the posterior distribution of the phylogenies for the best-fitting spatio-temporal diffusion model of each clade, in the form of a maximum clade credibility (MCC) trees. We then used FigTree^85^ to plot the resulting MCC trees colored by the inferred among-branch spatial diffusion rate variation for each clade. Finally, we used spreaD3^86^ to generate keyhole markup language (KML) files to visualize clades’ MCC trees, and then superimposed them on the resulting risk map of PRRSv outbreaks predicted by the final Maxent model using Google Earth Pro.

## Electronic supplementary material

Supplementary Information 

File S1

File S2

File S3

File S4
