# Supplementary material for: Novel approaches for Spatial and Molecular Surveillance of Porcine Reproductive and Respiratory Syndrome Virus (PRRSv) in the United States
Source: Sci Rep. 2017 Jun 28;7:4343. doi: 10.1038/s41598-017-04628-2 (PMC5489505; doi:10.1038/s41598-017-04628-2)
Supplement: Supplementary file 1 — Supplementary Information [file 41598_2017_4628_MOESM1_ESM.pdf]

Title page

Novel approaches for Spatial and Molecular Surveillance of Porcine Reproductive and Respiratory Syndrome Virus (PRRSv) in the United States

Moh A. Alkhamis [1,2]*, Andreia G. Arruda[3], Robert B. Morrison[1], Andres M. Perez [1]

[1]Department of Veterinary Population Medicine, College of Veterinary Medicine, University of Minnesota, St. Paul, USA

[2]Faculty of Public Heath, Health Sciences Center, Kuwait University, Kuwait

[3]Department of Veterinary Preventive Medicine, College of Veterinary Medicine, The Ohio State University, Columbus, USA

Supplementary Materials

Supplementary Table 1. Properties and sources of the enviromental predictors used to predict the high-risk areas for PRRSV outbreaks in the Midwest.

<table>
<tr><th>ID</th><th>Source</th><th>Time period</th><th>Spatial resolution</th><th>Type</th></tr>
<tr><td>1</td><td rowspan="15">WorldClim Global Climate Data</td><td rowspan="15">1960-1990</td><td rowspan="15">5 km^2</td><td>BIO1 = Annual Mean Temperature</td></tr>
<tr><td>2</td><td>BIO2 = Mean Diurnal Range (Mean of monthly (max temp - min temp))</td></tr>
<tr><td>3</td><td>BIO3 = Isothermality (BIO2/BIO7) (* 100)</td></tr>
<tr><td>4</td><td>BIO4 = Temperature Seasonality (standard deviation *100)</td></tr>
<tr><td>5</td><td>BIO5 = Max Temperature of Warmest Month</td></tr>
<tr><td>6</td><td>BIO6 = Min Temperature of Coldest Month</td></tr>
<tr><td>7</td><td>BIO7 = Temperature Annual Range (BIO5-BIO6)</td></tr>
<tr><td>8</td><td>BIO10 = Mean Temperature of Warmest Quarter</td></tr>
<tr><td>9</td><td>BIO11 = Mean Temperature of Coldest Quarter</td></tr>
<tr><td>10</td><td>BIO12 = Annual Precipitation</td></tr>
<tr><td>11</td><td>BIO13 = Precipitation of Wettest Month</td></tr>
<tr><td>12</td><td>BIO14 = Precipitation of Driest Month</td></tr>
<tr><td>13</td><td>BIO15 = Precipitation Seasonality (Coefficient of Variation)</td></tr>
<tr><td>14</td><td>BIO16 = Precipitation of Wettest Quarter</td></tr>
<tr><td>15</td><td>BIO17 = Precipitation of Driest Quarter</td></tr>
<tr><td>16</td><td>MODIS-based Global Land Cover</td><td>2014</td><td>0.5 km^2</td><td>Land cover with 16 discrete spatial features
0 = Water
1 = Evergreen Needle leaf Forest
2 = Evergreen Broadleaf Forest
3 = Deciduous Needle leaf Forest
4 = Deciduous Broadleaf Forest
5 = Mixed Forests
6 = Closed Shrublands
7 = Open Shrublands
8 = Woody Savannas
9 = Savannas
10 = Grasslands
11 = Permanent Wetland
12 = Croplands
13 = Urban and Built-Up
14 = Cropland/Natural Vegetation Mosaic
15 = Snow and Ice
16 = Barren or Sparsely Vegetated</td></tr>
<tr><td>17</td><td>USDA-Census of Agriculture website</td><td>2012</td><td>5 km^2</td><td>Latitude and Longitude of all pig farms in the US converted into a raster using a kernel density function</td></tr>
</table>

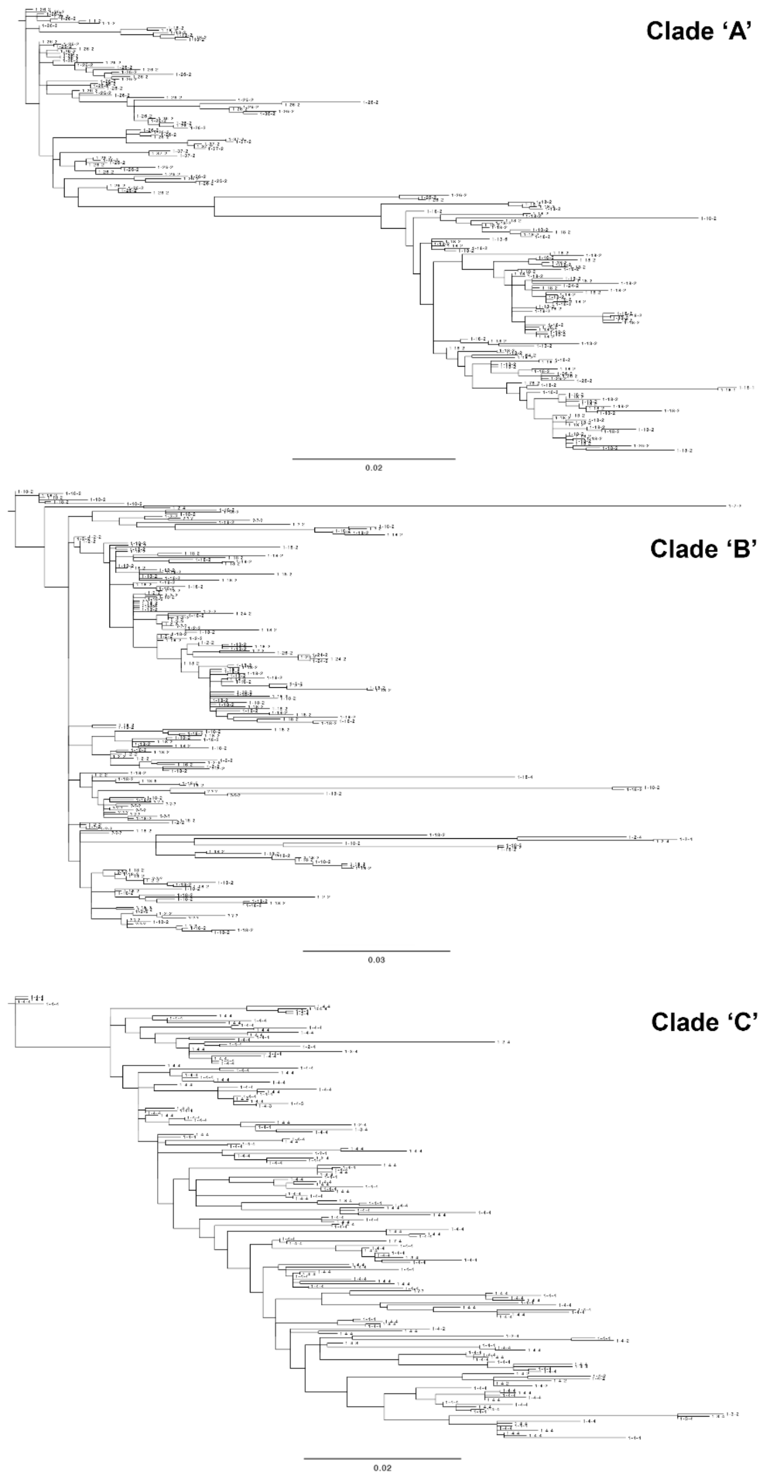

Figure S1. Maximum likelihood phylogeny of PRRSV selected clades’ (A, B and C) inferred from ORF-5 sequences collected between 1998 and 2016 in swine farms in the Midwest of U.S.

Table S2. Selected clades' Bayes factor s (BFs) comparisons of relaxed-clock demographic models using stepping-stone (SS) and path-sampling (PS) methods. BFs based on the average SS marginal likelihood estimates are on the upper off-diagonal of the table, while BFs based on the average PS marginal likelihood estimates are on the lower off-diagonal of the table. Best fitting parametric and non-parametric models are boldfaced.

	Bayes Factor									
	Clade A									
Relaxed-clock Model	UCED+CP	UCED+EG	UCED+EGx	UCED+LG	UCED+PCBS	UCLN+CP	UCLN+EG	UCLN+EGx	UCLN+LG	UCLN+PCBS
UCED[a]+CP[b]	—	-215.05	-59.98	488.29	-83.94	22.71	-56.34	-91.34	530.08	-69.5
**UCED+EGx[c]**	**165.4**	**—**	**155.07**	**703.34**	**131.11**	**237.76**	**158.71**	**123.71**	**745.13**	**145.55**
UCED+EG[d]	62.3	-103.1	—	548.27	-23.96	82.69	3.64	-31.36	590.06	-9.52
UCED+LG[e]	-488.1	-653.5	-550.4	—	-572.23	-465.58	-544.63	-579.63	41.79	-557.79
UCED+*BSg[f]	62.14	-103.26	-0.16	550.24	—	106.65	27.6	-7.4	614.02	14.44
UCLN+CP	-39.6	-205	-101.9	448.5	-101.74	—	-79.05	-114.05	507.37	-92.21
UCLN+EGx	30.5	-134.9	-31.8	518.6	-31.64	70.1	—	-35	586.42	-13.16
UCLN+EG	94.4	32.1	32.1	582.5	32.26	134	63.9	—	621.42	21.84
UCLN+LG	-530.5	-42.4	-592.8	-42.4	-592.64	-490.9	-561	-624.9	—	-599.58
UCLN+BSg	64.1	1.96	1.8	552.2	1.96	103.7	33.6	-30.3	594.6	—
	Clade B									
UCED+CP	—	-0.82	1.72	-10.28	3.04	1.16	-10.83	-36.23	-16.51	-14.94
UCED+EGx	15.5	—	2.54	-9.46	3.86	1.98	-10.01	-35.41	-15.69	-14.12
UCED+EG	4.2	-11.3	—	-12	1.32	-0.56	-12.55	-37.95	-18.23	-16.66
UCED+LG	18.2	2.7	14	—	13.32	11.44	-0.55	-25.95	-6.23	-4.66
UCED+BSg	-3.7	-19.2	-7.9	-21.9	—	-1.88	-13.87	-39.27	-19.55	-17.98
UCLN[g]+CP	5	-10.5	0.8	-13.2	8.7	—	-11.99	-37.39	-17.67	-16.1
UCLN+EGx	15.2	-0.3	11	-3	18.9	10.2	—	-25.4	-5.68	-4.11
**UCLN+EG**	**35.9**	**31.7**	**31.7**	**17.7**	**39.6**	**30.9**	**20.7**	**—**	**19.72**	**21.29**
UCLN+LG	9.3	-8.9	5.1	-8.9	13	4.3	-5.9	-26.6	—	1.57
UCLN+BSg	22.67	26.37	18.47	4.47	26.37	17.67	7.47	-13.23	13.37	—
	Clade C									
UCED+CP	—	-7.7	-61.6	-142.9	-77.1	-14.9	-27.9	-102.8	-108.3	-103.26
UCED+EGx	10.1	—	-53.9	-135.2	-69.4	-7.2	-20.2	-95.1	-100.6	-95.56
UCED+EG	61.8	51.7	—	-81.3	-15.5	46.7	33.7	-41.2	-46.7	-41.66
**UCED+LG**	**167.7**	**157.6**	**105.9**	**—**	**65.8**	**128**	**115**	**40.1**	**34.6**	**39.64**
UCED+BSg	50.45	40.35	-11.35	-117.25	—	62.2	49.2	-25.7	-31.2	-26.16
UCLN+CP	18.8	8.7	-43	-148.9	-31.65	—	-13	-87.9	-93.4	-88.36
UCLN+EGx	31.2	21.1	-30.6	-136.5	-19.25	12.4	—	-74.9	-80.4	-75.36
UCLN+EG	106.6	44.8	44.8	-61.1	56.15	87.8	75.4	—	-5.5	-0.46
UCLN+LG	142.5	-25.2	80.7	-25.2	92.05	123.7	111.3	35.9	—	5.04
UCLN+BSg	123.1	72.65	61.3	-44.6	72.65	104.3	91.9	16.5	-19.4	—

[a]Uncorrelated relaxed clock with exponential distribution
[b]Constant population size coalescent model
[c]Expansion population size coalescent model
[d]Exponential population size coalescent model
[e]Logistic population size coalescent model
[f]Bayesian Skygrid coalescent model
[g]Uncorrelated relaxed clock with log-normal distribution
*non-parameteric demographic model

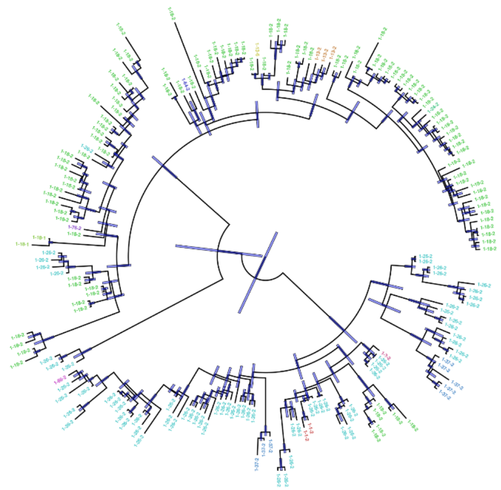

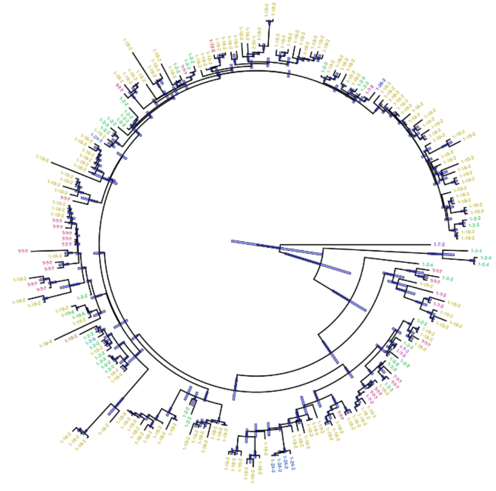

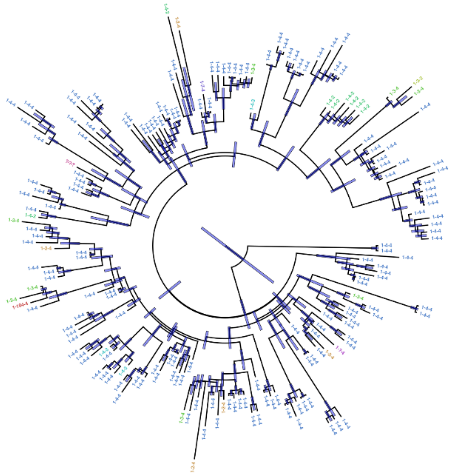

Figure S2. Maximum clade credibility (MCC) trees of PRRSV selected clades' (A, B and C) inferred from ORF-5 sequences collected between 1998 and 2016 in swine farms in the Midwest of USA. Branch lengths are rendered proportional to absolute time (Years). Nodes correspond to median ages and the blue horizontal bars at nodes represent the corresponding 95% HPDs for divergence-time estimates. Tips are labeled and colored by the isolates RFLP-types.

File S1. Alignment of PRRSV ORF-5 gene sequences (n = 3,582) in Fasta format used in this study.

File S2. Three-dimensional movie (in Quicktime Movie format) for Clade's A tree origin, spread and ecology in the Midwest from 2002 to 2016 onward. The figure is composed of satellite images captured as a movie from Google Earth Pro (https://www.google.com/earth/).The movie corresponds to Figure 5 in the main manuscript.

File S3. Three-dimensional movie (in Quicktime Movie format) for Clade's B tree origin, spread and ecology in the Midwest from 1991 to 2016 onward. The figure is composed of satellite images captured as a movie from Google Earth Pro (https://www.google.com/earth/).The movie corresponds to Figure 5 in the main manuscript.

File S4. Three-dimensional movie (in Quicktime Movie format) for Clade's C tree origin, spread and ecology in the Midwest from 2006 to 2016 onward. The figure is composed of satellite images captured as a movie from Google Earth Pro (https://www.google.com/earth/).The movie corresponds to Figure 5 in the main manuscript.
